# Systematic Review of Combined Pelvic Ring and Acetabular Injuries: What Do We Know From the Literature?

**DOI:** 10.7759/cureus.41843

**Published:** 2023-07-13

**Authors:** Hunter Ross, Sasha Stine, Kevin Blue, Trevor D Wolterink, Rahul Vaidya

**Affiliations:** 1 Orthopaedic Surgery, Wayne State University Detroit Medical Center, Detroit, USA; 2 Orthopaedic Surgery, Wayne State University School of Medicine, Detroit, USA

**Keywords:** anterior subcutaneous pelvic fixation, anterior fixation, anterior pelvic ring injury, pelvic fracture, pelvic ring injury, concomitant pelvic ring and acetabular fracture, combined pelvic ring and acetabulum fracture

## Abstract

The purpose of this review is to examine the literature on combined pelvic ring and acetabular fractures. We hope to further define the classifications, severities (ISS & Mortality), healing, radiographic parameters, and functional outcomes of such injuries to report all potential recommendations based on findings.

We used the Preferred Reporting Items for Systematic Reviews and Meta-Analyses (PRISMA) guidelines, and a systematic search on PubMed and Google Scholar was performed. Articles included were in the English Language or through English translation, between the years 1996 and 2022. Articles that had met the inclusion criteria were systematically assessed for the relevance of their content. Eleven articles were identified with a total of 985 patients. All eleven were retrospective case series and the presence of both an injury within the pelvic ring and another injury within the acetabulum, either ipsilateral or contralateral, was the indication of a combination injury.

The overall mortality rate averaged over all studies was 7.9% and the Injury Severity Score (ISS) of 22.98. When considering the higher mortality rate seen in pelvic ring injuries compared to the isolated acetabulum, there appears to be survivability beyond reductive means as a reason for reducing and fixing the pelvic ring first. However, accurate reduction of the acetabulum has a greater weight in overall patient recovery compared to the reduction of the pelvic ring and thus surgical emphasis on the anatomic reduction of the acetabulum may be paramount. Despite this good to excellent outcomes can be achieved with careful preoperative planning and surgical execution in patients with fractures of the pelvic ring and acetabulum. Further research as well as uniform radiographic scoring system and outcomes scores should be required to better evaluate and treat these injuries.

## Introduction and background

The combination of pelvic ring disruptions and acetabular fracture is infrequent based on all known recent reports, with an occurrence rate of 9% of all acetabular fractures and 5%-15.7% of all pelvic ring disruptions [1-5]. Combined ring and acetabulum fractures are usually the result of high-energy trauma with a distribution equally between anterior-to-posterior (AP) and lateral compression (LC) forces [1,2,5]. As described by Letournel, the acetabular fracture component of these combined fractures is typically complex [6]. Concomitant injuries of the pelvis and acetabulum are seen in patients with higher injury severity scores (ISS) and mortality rates above which are most commonly reported for isolated pelvic ring disruptions [1-3]. To date, there are few reports of either disruption of the pelvic ring or the fracture of the acetabulum that have included the resulting combination injury scenarios within their studies, and even fewer have directly or indirectly reported the outcomes of said scenarios after the treatment of such injuries [3,5].

In addition to the lack of literature, there yet remains some controversy and misperception over what constitutes the combination of injuries. Some pelvic fractures include bilateral, high pubic root or ramus injuries, which may even involve the anterior acetabular articular surface with minimal displacement and/or have little bearing on acetabular function [5]. These fractures oftentimes are referred to as either high-root ramus fractures or low-anterior column acetabulum fractures. It is important to distinguish between these two entities as separate and distinct given the treatment algorithms may differ. In addition, some acetabular fractures extend into the sacroiliac (SI) joint yet both situations may have little bearing on actual outcome [7]. Within this article, we will confirm the understanding that high-root ramus fractures do not involve the acetabular articular surface and are classified in the pelvic ring injury pattern whereas low anterior acetabulum fractures do involve the articular surface and are included in the classic elementary patterns as outlined by Letournel [6]. Combined pelvic ring and acetabulum literature including management protocols is sparse and there is an observable lack of extensive studies on the matter. Due to the existing literature, it was our feeling that universal treatment recommendations remain vague, with multiple uncertain protocols in place, and that patient outcomes were yet undetermined.

The intent of this review is to examine the literature on combined pelvic ring and acetabular injuries. We hope to further define the classifications, severities (ISS & Mortality), healing, radiographic parameters, and functional outcomes of such injuries to report all viable recommendations based on findings.

## Review

A systematic review of the literature was performed according to the methods described within the Preferred Reporting Items for Systematic Reviews and Meta-Analyses (PRISMA) statement. We also completed a search and registered the study with the international database PROSPERO to ensure no other studies were currently ongoing (Study ID 384830). Studies included within this study were original articles that were pertinent to our research questions, published in the English Language or through English translation, between the years 1996 and 2022.

**Figure 1:**
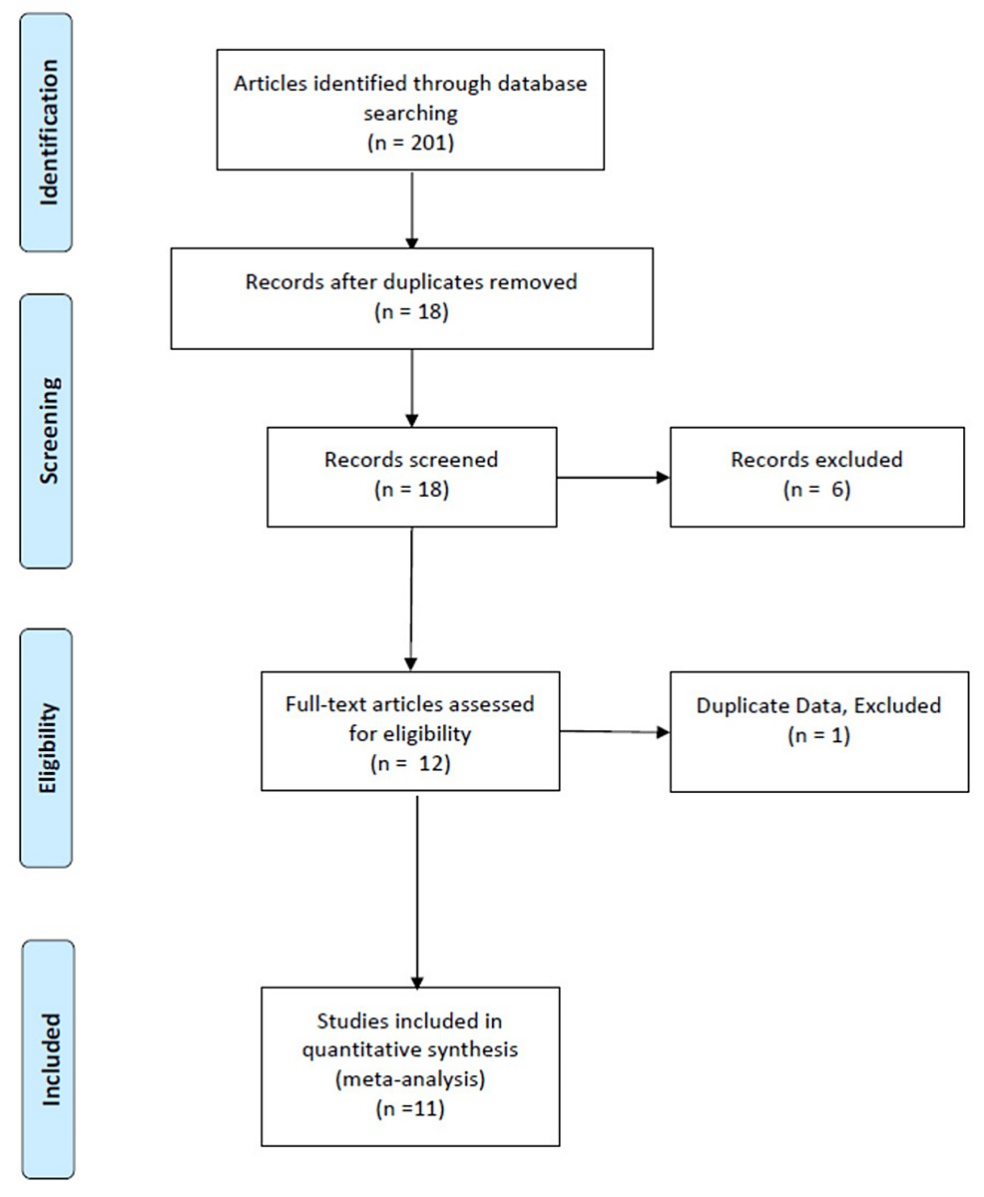
Flow diagram.

Medical search terms were used with the strings “concomitant pelvic ring and acetabular fracture”, “combined pelvic ring and acetabular fracture”, the substitution of “fracture” as “injury” and “pelvic ring” with “pelvis”, “combined fracture of the pelvic ring and acetabulum”, the substitution of “fracture” as “injury” and “pelvic ring” with “pelvis”, the exclusion of the search parameters “concomitant” and “combined” with both uses of “fracture” and “injury”, “simultaneous fracture of the pelvic ring and acetabulum”, and finally the substitution of “fracture” as “injury” and “pelvic ring” with “pelvis.” These terms were searched within PubMed, Google Scholar, and the Wayne State University (WSU) Library Database. Databases and articles were accessed through Wayne State University (WSU) Library, Detroit, Michigan. Articles that had met the inclusion criteria were systematically assessed for inclusion (Figure 1). Initially, titles were screened for primary inclusion and exclusions then all abstracts obtained were further assessed for eligibility. The full articles which met the relevance and inclusion criteria were obtained and then reviewed, giving specific consideration to the importance of our topic. References to the full texts were also reviewed to discover any other relevant studies that were missed in our initial review of the literature. The final list of studies relevant to our topic was reviewed according to their study designs, analyses, and interpretations, as well as the validity of their results.

After the application of our defined eligibility criteria, a total of 18 studies were identified [1-18]. One study was removed from our collected data due to duplicate data and six were removed as they did not meet inclusion criteria. After initial exclusion, 11 articles remained with a total of 985 patients [1-4,7-13]. Of the 11, all were retrospective case series and the presence of both an injury within the pelvic ring and another injury within the acetabulum was the indication of a combination injury.

For pelvic injury, three studies used Young & Burgess to classify injury [1,12-14], one listed injury without classification [10], three used both Young & Burgess and AO/OTA classification for the pelvis [2,7,13], and one used the Tile classification [3]. For acetabular injury, six used the Judet and Letournel classification [1,3,10-13], one used AO/OTA classification for the acetabulum [2], one used both Judet and Letournel and AO/OTA classification for the acetabulum [7], and three did not provide specific classification [4,8-9]. When possible, acetabular fractures were reclassified from Judet and Letournel to AO/OTA classification of acetabulum [10-11,13]. Of the 985 patients, 450 patients had an average Injury Severity Score (ISS) of 22.98 [1-2,7-8,11-13] Average ISS was calculated by the multiplication of each article’s individual sum cases by their ISS, adding the individual sums of all seven studies and then dividing by the total sum of 450 patients to collect the average of seven studies. From six studies with a total of 959 patients, 453 patients had a recorded mortality rate of 7.9% [1-2,7-8,9,12]. The calculation for the average mortality rate was done by multiplication of each article’s individual sum cases by their mortality rates, adding each individual sum from all six studies, and then dividing the combined sum by the total number of patients (Table 1).

**Table 1:** Data. APC = anteroposterior compression, LC = lateral compression, NR = not reported, OTA = Orthopaedic Trauma Association, CM = combined mechanism, VS = vertical shear. OU = other unilateral. OB = other bilateral.

Table 1							
Recent Studies on Combination of Pelvic Ring Disruptions and Acetabulum Fractures							
Study	No. of Patients with Combined Injuries	Average Age in Years (Range)	Young and Burgess Classification (%)	AO/OTA Classification (%)	Acetabular Classification (%)	Average Injury Severity Score (Range)	Mortality Rate (%)
Osgood et al. [1]	40	40 (16-79)	LC1, 32; LC2, 7; LC3, 7; APC2, 48; APC3, 4; VS, 2	61B2.1, 32; 61B2.2, 7; 61B2.3, 48; 61B3.1, 7; 61C1, 7	62A1, 5; 62A3.1, 2; 62A3, 18; 62B1, 27; 62B2, 23; 62B3, 11; 62C, 14	27.9 (4-59)	13
Suzuki et al. [2]	82	36.7 (13-95)	LC, 46; APC, 48; VS, 6	61B1, 28; 61B2, 28; 61B3, 7; 61C1, 26; 61C2, 6; 61C3, 5	OTA: Transverse, 61.2; Non-Transverse, 38.8	29.3 (9-66)	5
Cai et al. [3]	21	43.2 (21-55)	NR	62A2, 14; 62A3, 10; 62B1, 52; 62C, 24	62A2.3, 14; 62A3, 10; 62B1, 52; 62C, 24	NR	NR
Gänsslen et al. [4]	401	NR	NR	NR	Simple, 52.2; Complex, 47.8: [Bilateral: 6.2]	NR	NR
Vaidya et al. [7]	38	37.5 (16-79)	LC1, 3; LC2, 15; LC3, 21; APC2, 13; APC3, 13; Bi-LC2, 3; Bi-APC2, 24; Bi-APC3, 5; VS-APC2, 3	61B2.1, 3; 61B2.2, 15; 61B2.3, 13; 61B3.1, 18; 61B3.2, 3; 61B3.3, 24; 61C1.2, 10; 61C2.2, 3; 61C2.3, 3; 61C3.1, 8	62A1.1, 5; 62A3.1, 10; 62A3.2, 3; 62A3.3, 3; 62B1.2, 18; 62B2.2, 21; 62B3.1, 5; 62B3.2, 3; 62B3.3, 3; 62C1, 10; 62C2, 16; 62C3, 3	20 (4-57)	8.3
Tibbs et al. [8]	188	NR	NR	NR	NR	17.5 (7-28)	10.6
Dalal et al. [9]	63	NR	NR	NR	N/A	NR	1.5
Selek et al. [10]	51	37.6 (19-63)	Ipsilateral, 18; Contralateral, 11; Bilateral Sacroiliac Separations, 3; Sacrum Fractures, 2	NR	62B2, 14; 62B1 a2, 20; 62B2, 7	NR	NR
Hess et al. [11]	33	37.3 (31.6-43)	LC1, 6; LC2, 3; LC3, 12; APC2, 49; APC3, 3; CMI, 12; BR, 15	NR	62A3, 9; 62A2, 3; 62A1, 6; 62B2, 15; 62B1 a2, 25; 62B2, 18; 62B3, 3; 62C, 12 OU, 3; OB, 6	30 (26-34)	NR
Porter et al. [12]	42	NR	LC, 48; APC, 42; VS, 3; CM, 7	NR	62A1, 7; 62A3, 21; 62A2, 2; 62B1.2b, 29; 62B1, 29; 62B2, 5; 62B3, 5; 62C, 2	30 (9-66)	6.1
Yu et al. [13]	26	47.8 (33-63)	NR	61A1.1, 1; 61B2.1, 2; 61B2.2, 10; 61B2.3, 3; 61B3.2, 1; 61B3.3, 2; 61C1.3, 4; 61C3.1, 2; 61C3.3, 2	62A3, 10; 62B1, 5; 62B2, 3; 62B3,4; 62C, 5	16.7 (8-25)	NR

Vaidya et al. reviewed 174 patients who sustained combined pelvic ring and acetabulum fractures over a 13-year period and reported their radiographic and clinical outcomes. Of the 174 combined injuries, those excluded from their study were fractures of either pelvic ring or acetabulum that were fixed but not both fixed, were not treated according to their protocol, or were lost to follow-up. Thus 39 patients with 41 acetabular injuries (two bilateral) were included in their study. Good to excellent outcomes were reported in 78% of patients and poor outcomes in 22%. It was noted by the authors that the reason for poor outcomes in the 22% included poor acetabular reduction, severe heterotopic ossification, sciatic nerve palsy that became persistent, and lastly, infection. Cai et al. in their study were able to achieve good to excellent reduction in 77% of their patients with excellent-good according to the Matta scoring method whilst noting the limitations of a small sample size. Yu et al. similarly reported outcome scores of 95% excellent pelvis and 65% excellent acetabulum scores using the Matta scoring method. Suzuki et al. achieved an anatomic reduction in 28 patients (41.2%), an imperfect reduction in 31 (45.6%), and a poor reduction in 8 (11.8%) utilizing Matta’s scoring. Gansslen et al. utilized Matta scoring and reported anatomic in 84.4% of type B injuries and imperfect reduction in 12.8%. Of the 245 type C injuries, they reported anatomic reductions in 56.7% and imperfect reduction in 6.9% indicating type C injuries confer more difficult to achieve anatomic reduction. Majeed outcomes scores were reported in only two studies [3,7,16].

**Figure 2:**
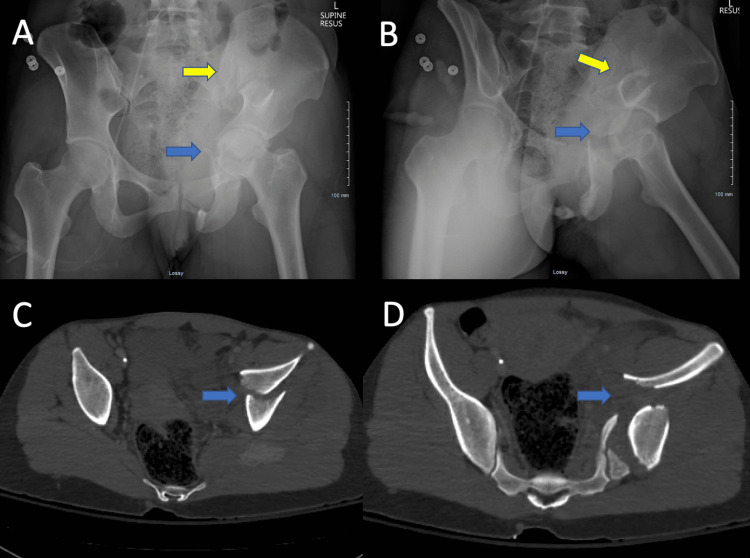
An example of an associated both column acetabulum fracture with sacroiliac joint involvement (combined pelvic ring and acetabulum fracture). Yellow arrows highlight the pelvic ring fracture. Blue arrows highlight the acetabulum fracture.

**Figure 3:**
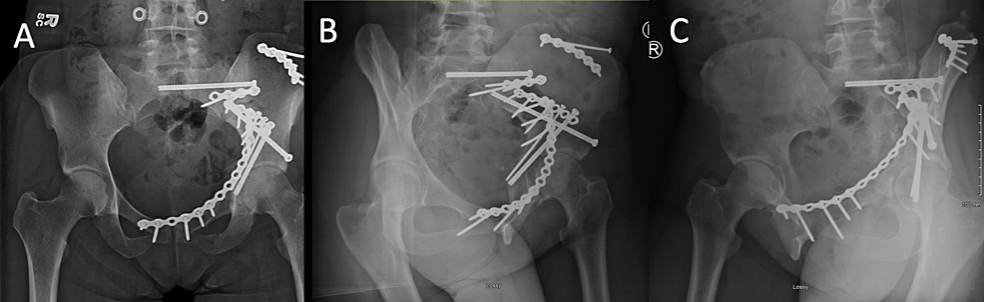
Fixation of the combined pelvic ring and acetabulum fracture in figure 2 proceeded from posterior (SI joint) to anterior with reduction and internal fixation placed through an ilioinguinal approach.

Our systemic review found 11 retrospective studies which included 985 patients. It is understood that combined pelvic ring and acetabular fracture patients first need to be evaluated according to the ATLS protocol with high importance on the treatment of patients who present with hemodynamic instability, pelvic sheeting or binder placement, and identify associated injuries. As it is generally accepted that the isolated disruption of the pelvic ring results in a higher mortality rate than the isolated fracture of the acetabulum (1.5%-13%), it may then be more logical to begin fixation of the pelvic ring [1-2,7-8]. According to Cai et al., Halvorson et al., and Vaidya et al., the choices of surgical approach, order of fixation, and even the methods for optimal treatment of combined pelvic ring and acetabular injuries remain uncertain and controversial [2,5,7]. In cases of combined injury of the pelvic ring and acetabulum, it is recommended by Vaidya et al., suggested by Cai et al. and Yu et al. that the treatment of the pelvic ring, specifically the posterior pelvic ring, should be performed before treatment of subsequent acetabular injuries (Figures 2, 3). In addition, Vaidya, Cai, Yu, and Halvorson et al. offer recommendations for an overall posterior-to-anterior approach to the overall concomitant injury scenario. This can be completed through any approach, anterior or posterior, dependent upon the fracture reduction proceeds in a posterior to anterior direction. In cases of fractures of the posterior wall, Cai et al., Halvorson et al., and Vaidya et al. recommend the Kocher-Langenbeck approach to reduce and secure the posterior wall as this is difficult or accurately fix through other approaches. In patients who exhibit sacroiliac joint diastasis or iliac wing fracture displacement, Cai et al. ascribe an ilioinguinal approach to the lateral window in order to view and secure the sacroiliac joint dislocation and/or reduce and stabilize the displaced iliac wing fracture and then under direct vision perform fracture reduction and fixation of the acetabulum. It appears that when considering the higher mortality rate seen in pelvic ring injuries compared to the isolated acetabulum, there is value in the fixation of the pelvic ring first prior to acetabulum fixation.

In a review by Halvorson et al., they state that the accurate reduction of the acetabulum has a greater weight in overall patient recovery compared to the reduction of the pelvic ring and that their surgical emphasis is the anatomic reduction of the acetabulum [3,5]. More recently, Yu et al. discussed their treatment strategy in which using Matta’s criteria for pelvic ring fractures may be useful for predicting the risk of subsequent inadequate reduction of the acetabulum indicating that reduction must be excellent before moving on to acetabulum reduction and fixation [13]. Despite these authors utilizing anterior approaches (ilioinguinal, anterior intrapelvic, and para rectus), their reduction sequence did not differ and they insured posterior ring fixation was completed through or in conjunction with this approach. What is different in Yu et al. protocol is they sought to seek and define anatomic reduction using Matta’s criteria of the pelvic ring before reducing and fixing the acetabulum. In a similar fashion, Selek et al. found that combined transverse-oriented acetabulum fractures with residual sacroiliac joint separation of >0.5 cm are likely to result in an unsatisfactory reduction of acetabulum fractures. Thus, it seems critical to have an anatomic or excellent reduction of the posterior pelvic ring prior to fixation of the acetabulum.

**Figure 4:**
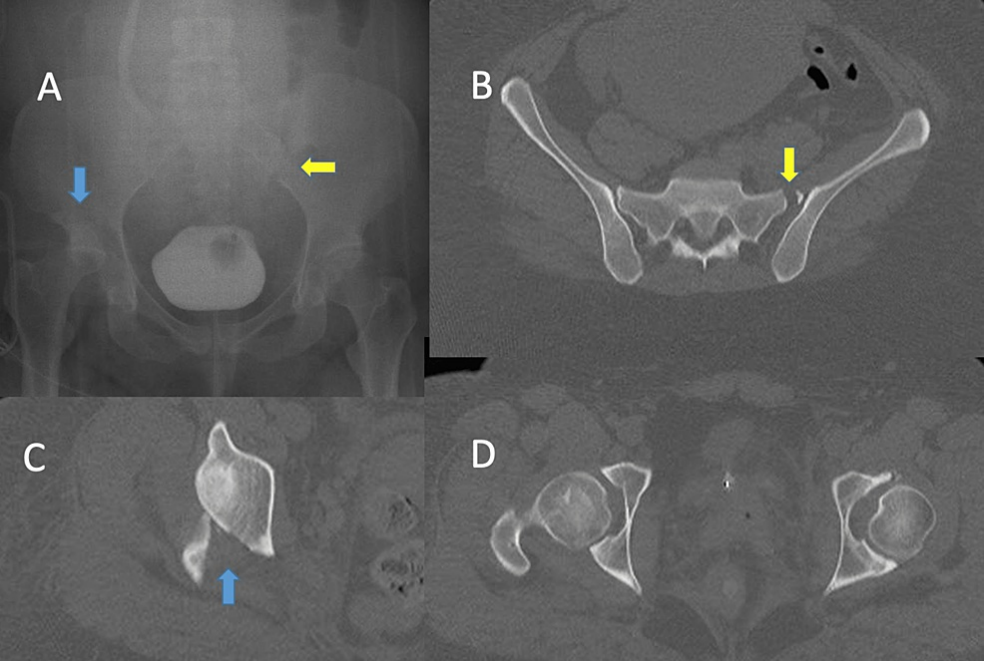
An example of a combined pelvic ring and acetabulum fracture. Yellow arrows highlight the pelvic ring fracture. Blue arrows highlight the acetabulum fracture.

**Figure 5:**
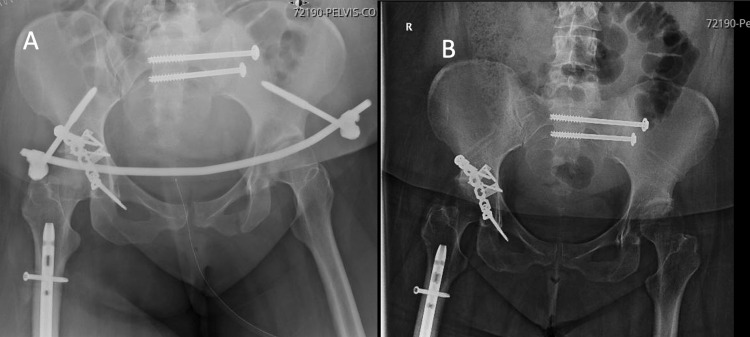
Stabilization of the combined pelvic ring and acetabulum fracture in Figure 4 began with posterior fixation first followed by flexible anterior fixation utilizing an INFIX and finalized with acetabular stabilization through a posterior Kocher-Langenbeck approach (A). Final x-rays after INFIX removal (B).

There is lacking clear treatment protocols for combined pelvic ring and acetabulum injuries. In comparison to other studies, Vaidya et al. outline a treatment protocol wherein the acetabulum fracture is evaluated to be a factor of the pelvic ring injury (i.e. associated with both columns) or with anterior column fracture exiting above the anterior inferior iliac spine (AIIS) [7]. For those fractures, these authors would work back to front considering each iliac wing separately and connecting these through an internal fixation (INFIX) device. The acetabulum fractures can then be addressed. While the posterior pelvic ring was reduced and secured, the anterior ring is held secured but flexible using an INFIX. Utilizing stiff or rigid fixation in the anterior portion of the pelvis (i.e. plate) before fixing the ilium or posterior column fracture can lead to difficulty with reduction, this is not affected with INFIX because the front remains somewhat mobile (Figures 4, 5).

In accordance with Cai et al., their treatment algorithm is to first perform a reduction followed by fixation of the posterior pelvis, then treat the acetabulum, and lastly reduce and fixate the anterior pelvic ring fractures [3]. These authors further elaborate that every attempt was made at the percutaneous fixation of the sacroiliac (SI) joint or sacral fractures. Regardless of the injury, the posterior pelvic ring requires anatomic reduction before moving to the acetabulum. Finally, the anterior pelvic ring can be fixed in Tile B pelvic ring injuries to restore stability if required. Furthermore, these authors consider that most acetabular fractures are capable of being treated via a singular anterior approach, and, therefore, recommend preparation for the potential need for a combined (anterior and posterior) approach [3].

Another advantage of posterior-to-anterior fixation is appreciated after reviewing treatment for failed fixation of the acetabulum and pelvic ring fractures. While only two of the eleven articles report complications after combined pelvic ring and acetabulum fracture fixation, there are no articles that review long-term follow-up and outcomes. It is reported that total hip arthroplasty as a treatment in patients with failed progressive arthritis or avascular necrosis after acetabulum fracture fixation surgery provides a reliable option with satisfactory outcomes [17]. If we then compare outcomes of pelvic ring fractures, Gerbershagen et al. found at a median follow-up of 52 months, 63.8% of patients reported chronic posttraumatic pelvic pain, with the highest prevalence in Tile B and C patterns (67% and 90% respectively) [18]. These findings may give further support to obtaining an anatomic reduction of the pelvic ring before reducing and fixing the acetabulum.

There are several limitations of this study including that the literature reports only retrospective reviews and very few studies have uniform reporting of outcomes and measurements. The assessment of study quality is thus limited. Furthermore, retrospective data collection is dependent on the accuracy of the collected and reported data. There are also heterogeneous study populations and injury mechanisms present. Radiographic parameters were reported in only five studies and complications were reported in two studies.

## Conclusions

Patients with combined pelvic ring and acetabulum fractures represent challenging injury patterns as well as patient presentations. Patient resuscitation needs to be the primary focus followed by stabilization of the pelvic ring and acetabulum. It appears the choice of the sequence of fixation is yet to be confirmed however from this review optimal results are achieved with a posterior to anterior reduction sequence whereby anatomic reduction of the posterior pelvic ring is mandatory before attempting acetabulum fixation and finally anterior fixation. We believe this stepwise approach to combined pelvic ring and acetabulum fractures leads to optimal results as indicated by the current body of research. Good to excellent outcomes can be achieved with careful preoperative planning and surgical execution in patients with fractures of the pelvic ring and acetabulum. Further research as well as uniform radiographic scoring system and outcomes scores should be required to better evaluate and treat these injuries.
